# ePRO symptom follow-up of colorectal cancer patients receiving oxaliplatin-based adjuvant chemotherapy is feasible and enhances the quality of patient care: a prospective multicenter study

**DOI:** 10.1007/s00432-023-04622-4

**Published:** 2023-02-21

**Authors:** Sanna Iivanainen, Ravi Ravichandra, Antti Jekunen, Reetta Arokoski, Santeri Mentu, Laura Lang, Jussi Ekström, Henri Virtanen, Vesa Kataja, Jussi P. Koivunen

**Affiliations:** 1 Department of Oncology and Radiotherapy, Oulu University Hospital and MRC Oulu, P.B. 22, 90029 Oulu, Finland; 2 Department of Oncology and Radiotherapy, Vaasa Central Hospital, Vaasa, Finland; 3 Kaiku Health Oy, Helsinki, Finland; 4 Department of Oncology and Radiotherapy, Turku University Hospital, Turku, Finland

**Keywords:** ePRO, CIPN, Chemotherapy, CRC, Adverse event, Adjuvant therapy

## Abstract

**Purpose:**

Electronic (e) patient-reported outcomes (PROs) have been shown to improve the quality of life and survival in chemotherapy treated advanced cancer patients. We hypothesized that multidimensional ePRO centered approach could improve symptom management, streamline patient flow, and optimize the use of healthcare resources.

**Methods:**

In this multicenter trial (NCT04081558), colorectal cancer (CRC) patients receiving oxaliplatin-based chemotherapy as adjuvant or in the first- or second-line setting in advanced disease were included in the prospective ePRO cohort, while a comparative retrospective cohort was collected from the same institutes. The investigated tool consisted of a weekly e-symptom questionnaire integrated to an urgency algorithm and laboratory value interface, which generated semi-automated decision support for chemotherapy cycle prescription and individualized symptom management.

**Results:**

Recruitment to the ePRO cohort occurred 1/2019–1/2021 (*n* = 43). The comparator group (*n* = 194) consisted of patients treated in the same institutes 1–7/2017. The analysis was limited to adjuvant treated (*n* = 36 and *n* = 35). The feasibility of the ePRO follow-up was good with 98% reporting easy usage and 86% improved care, while health care personnel valued the easy use and logical workflow. In the ePRO cohort, 42% needed a phone call before planned chemotherapy cycles, while this was 100% in the retrospective cohort (*p* = 1.4e−8). Peripheral sensory neuropathy was detected significantly earlier with ePRO followed (*p* = 1e−5) but did not translate to earlier dose reduction, delays, or unplanned therapy termination compared to the retrospective cohort.

**Conclusion:**

The results suggest that the investigated approach is feasible and streamlines workflow. Earlier symptom detection may improve the quality in cancer care.

## Introduction

Colorectal cancer (CRC) represents a major public health problem accounting for over 1 million cases of new cancers and about half a million deaths worldwide (Kerckhove et al. 2017). Even with curative surgery in those presenting the disease early, the risk of recurrence is significantly high. In colon cancer, oxaliplatin-based chemotherapy is the principal adjuvant therapy for patients with stage III and high-risk stage II disease as well as the backbone of treatment in metastatic and locally advanced settings (Argilés et al. 2020; Benson et al. 2021). Peripheral sensory neuropathy is one of the most serious adverse effects (AE) of oxaliplatin and up to 90% of patients may suffer from this (Hersman et al. 2016). Chemotherapy-induced peripheral neuropathy (CIPN) may result in persistent symptoms leading to the deterioration of quality of life (QoL) in cancer survivors. In a systematic review and meta-analysis consisting of 4179 adult cancer patients, CIPN affected 60.0% of patients at three 3 months, and 30.0% at six  months or more (Benson et al 2021). CIPN may potentially cause long-term effects on activities of daily living, concerning 47% of patients after years since the end of treatment (Seretny et al. 2014).

Electronic (e) patient-reported outcome (PRO) monitoring has been shown to improve the QoL and survival, and reduce the number of unscheduled visits in chemotherapy treated advanced cancer patients (Basch et al. 2015). Studies on digital symptom monitoring among the adjuvant chemotherapy therapy treated are scarce (Osborn et al. 2020; Moradian et al. 2018; Maguire et al. 2021) and it is unknown whether similar benefits can be achieved as seen in advanced settings. The life expectancy among the adjuvant treated is substantially longer and prevention of long-term AEs is likely to have a bigger impact on cumulative QoL. Digital approaches in healthcare can potentially improve accessibility and increase the comprehensiveness of care and streamline the processes in a cost-effective manner. Digital tools are especially convenient in facilitating communication independent of time and place, and duties requiring repeated numerical comparison (Holch et al. 2017; Kotronoulas et al. 2014; Lizée et al. 2019).

We speculated that a decision support tool integrating ePRO monitoring, personalized symptom management, and laboratory interface could have positive synergist impact on care pathway by improving patient follow-up, patient empowerment, and simplifying workflow. We selected CRC patients receiving oxaliplatin-based therapy as the pilot population due to the numerically large indication and potential long-term adverse events in the form of CIPN.

## Materials and methods

### Patients

We initiated a one-arm multicenter prospective clinical trial (ECHO) investigating the use of novel Kaiku Health ePRO tool in cancer care. The most important inclusion criteria were CRC planned to receive oxaliplatin-based chemotherapy as an adjuvant therapy or in the first- or second-line setting of advanced disease, age ≥ 18 years, ECOG performance score of 0–2, and internet access. The study end points included number of phone calls related to prescribing a new chemotherapy cycle per patient or treatment cycle, unscheduled outpatient and inpatient visits, development of peripheral neurotoxicity, the number of chemotherapy dose reductions, and dose delays. Patients’ perspectives were collected via monthly e-questionnaires developed by the study team and used in the previous studies. Perceptions of health care personnels (HCPs) involved in the study were collected through a one-time semi-structured interview.

The patient recruitment occurred 7/2019–1/2021 and the last date of follow-up was 5/2022. A retrospective comparative cohort was collected from the same institutes including all the CRC patients initiating oxaliplatin-based adjuvant therapy or chemotherapy treatment in the first- or second-line setting of advanced disease between 1-7/2017.

### ePRO tool

Kaiku Health ePRO follow-up module consists of a questionnaire of 17 symptoms which assess both the presence and severity of the symptom (blood in urine, dysuria, eye symptoms (decreased vision/other), peripheral sensory neuropathy, pain, constipation, cough, decreased appetite, diarrhea, fatigue, fever, mouth sores, nausea, rash/skin changes, shortness of breath, vomiting). The symptoms selected for the Kaiku Health symptom tracking tool for cancer medical treatment include typical side effects of chemotherapy. The questions for each symptom in the instrument were developed based on NCI-CTCAE by converting the description of gradings into a patient-friendly language. Besides recording a presence of a symptom, severity algorithm that grades the symptom according to NCI-CTCAE was applied. The severity algorithm triggered an email alert to the study physician of the care unit with preset limits (presence of a grade 3 or higher symptom or a rise of a symptom severity from grade 0 to 2). The patients were informed that the care unit reacts to the alerts promptly within 3 days; thus, the ePRO follow-up is intended only for non-urgent communication, and, in critical matters, patients were adviced to contact the emergency care. In addition, patients received tailored, evidence-based personalized self-care advice according to e-symptoms and their grade. The tool also included a messaging option through which patients could communicate with their care team directly.

### Study approvals

The study was approved by the ethics committee of Northern Ostrobothnia Hospital District (study no: 273/2017) and registered in an international clinical study registry (NCT04081558). The study was carried out in accordance with the principles of the Declaration of Helsinki. All patients provided written informed consent.

### Statistical analysis

Fischer’s exact test was used to compute statistical significance in binary variables. Kaplan–Meier method using log-rank test was utilized for time-dependent end points. Continuous end points were calculated from the date of first chemotherapy to the event or end-of follow-up. The results were presented with 95% confidence level. *P* values < 0.05 were regarded as statistically significant.

## Results

### Patient population

**Fig. 1:**
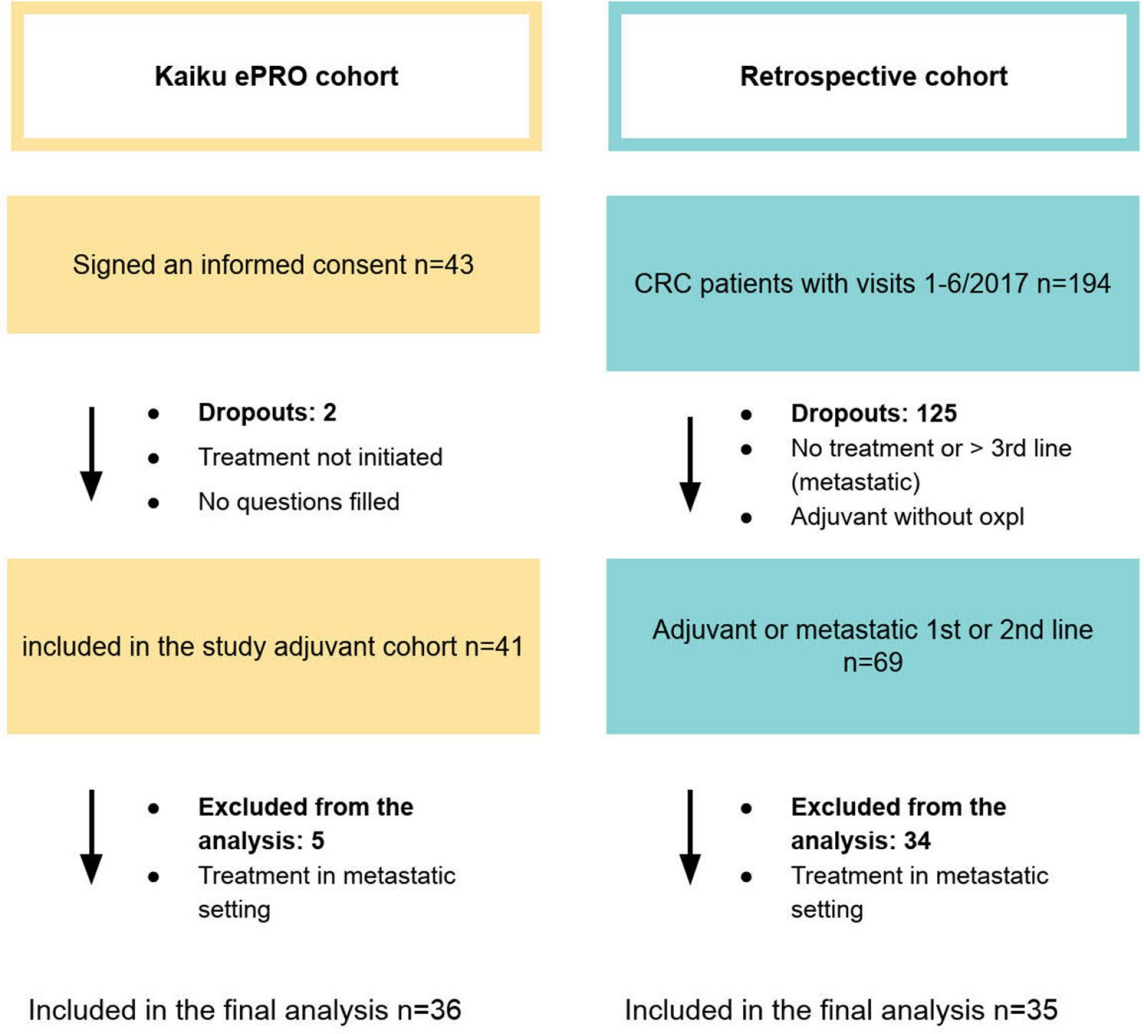
Patient accrual for the prospective ePRO and retrospective cohorts

The patient recruitment to the prospective ePRO cohort occurred 1/2019–1/2021 (*n* = 43), while the retrospective comparator group consisted of CRC patients (*n* = 194) initiating the treatment in the same institutes 1–7/2017. Since only a limited number of patients with metastatic disease were recruited in the prospective study (*n* = 4), we limited the final analysis only to the patients treated in adjuvant setting (prospective *n* = 36; retrospective *n* = 35). Flowchart of patient accrual and analysis is presented in Fig. 1.

The demographics of the both cohorts are presented in Table 1. In brief, cohorts were sufficiently alike with 66 vs. 60% of the subjects’ males (ns), median age 62 vs. 66 years (ns), and ECOG 0 in 11% vs. 47% (*p* = 0.001). In the retrospective cohort, the reduction rate of any of the chemotherapy components dose by < 10% of the recommended was higher with 19% vs 40% (ns).

**Table 1:** Patient characteristics

	ePR * n* (%)	Retrospective *n* (%)	Significance
	36 (100)	35 (100)	
Age (median)	62	66	NS
Sex			
Male	24 (66)	21 (60)	NS
Female	12 (34)	14 (40)	
ECOG			
0	4 (11)	16 (47)	*p* = 0.001
≥ 1	32 (89)	18 (53)	
Reduced initial dose (> 10%)			
No	29 (81)	21 (60)	NS
Yes	7 (19)	14 (40)	
Stage			
2	4 (11)	9 (25)	NS
3	25 (69)	23 (66)	
Unknown	7 (19)	3 (9)	

### Feasibility of the integrated digital symptom monitoring and laboratory interface

**Fig. 2:**
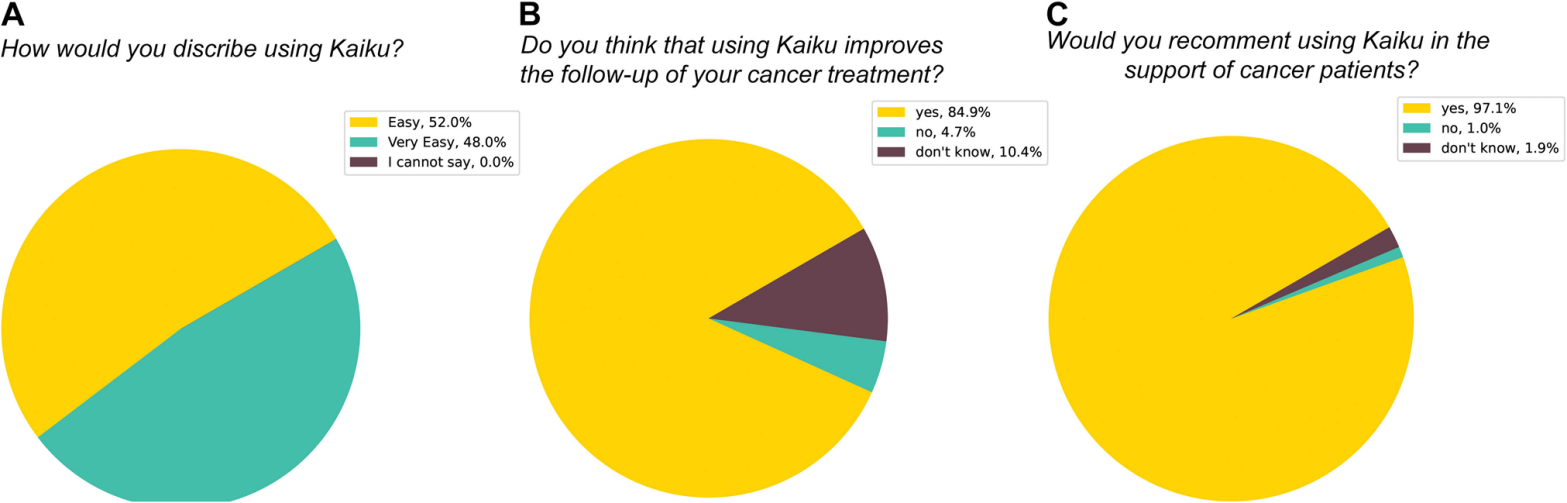
Patient experience in the ePRO cohort. The median answers to patient experience survey (**A**–**C**)

The feasibility of the ePRO follow-up was found to be good with 98% of the patients reporting that the use of the platform was very easy or easy to use, while only 0.6% said that usage was hard. Majority (86%) of the patients felt that the tool improved their cancer care, and 99% would recommend the platform to others (Fig. 2). Three HCPs involved in the study were interview in semi-structured fashion. HCPs valued the most easiness of use and logical workflow as well as messaging option, reduced phone calls, and improved patient access and satisfaction (not shown).

### Use of healthcare resources

Utilization of healthcare resources was investigated between the prospective ePRO and retrospective comparator cohorts. Among ePRO-patients, only 42% of the subjects needed a phone call before any of the planned chemotherapy cycles, while this was 100% in the retrospective cohort (*p* = 1.4e−8). In both cohorts, the number of additional outpatient or inpatient visits during the chemotherapy course was registered in four patients (11%) in both categories and cohorts (ns) (Table 2).

**Table 2:** Number of patients with phone calls before a new cycle and additional inpatient and outpatient visits during the first 90 days

	ePRO *n* (%)	Retrospective *n* (%)	Significance
	36 (100)	35 (100)	
Patient with phone calls			
Yes	15 (42)	35 (100)	1.40E−08
No	21 (58)	0 (0)	
Outpatient visits			
Yes	4 (11)	4 (11)	NS
No	32 (89)	32 (89)	
Inpatient visits			
Yes	4 (11)	4 (11)	NS
No	32 (89)	32 (89)	

### Occurrence of symptoms, chemotherapy dose reduction, dose delays, or unplanned treatment termination

**Fig. 3:**
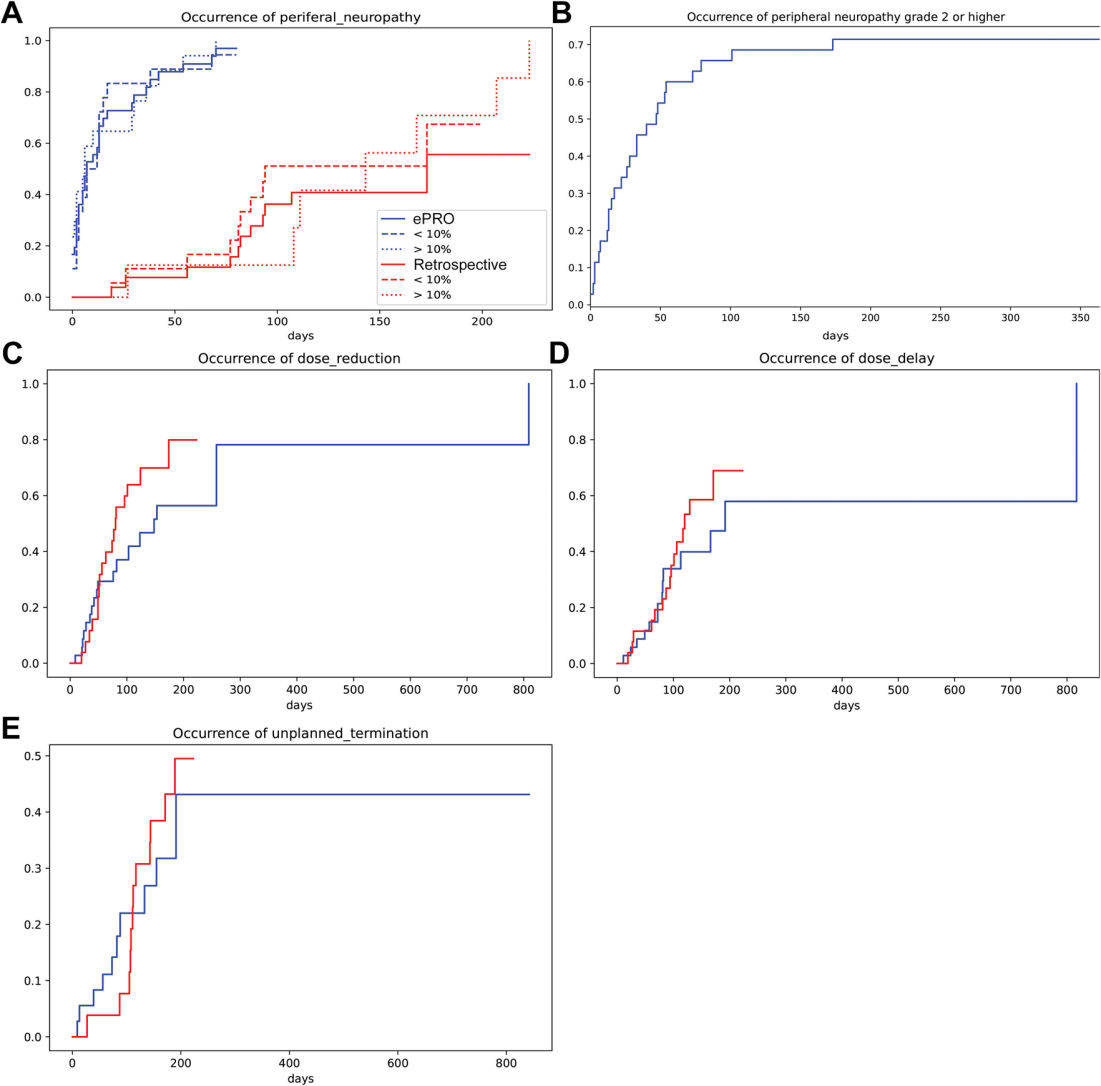
The occurrence of peripheral sensory neuropathy, chemotherapy dose reductions, dose delays, or unplanned treatment termination. **A** The occurrence of peripheral sensory neuropathy in ePRO (blue) and retrospective (red) cohorts in all (continuous) or according to initial chemotherapy dose (dotted, > 10% vs. ≤ 10% reduced dose). **B** The occurrence of grade ≥ 2 peripheral sensory neuropathy in the ePRO cohort. **C** The occurrence of chemotherapy dose reductions. **D** The occurrence of chemotherapy dose delays. **E** The occurrence of unplanned chemotherapy treatment termination

The occurrence of peripheral sensory neuropathy was analyzed between cohorts. Any grade peripheral sensory neuropathy occurred significantly earlier among the ePRO followed (*p* = 1e−5) with a median time of 7 days to occurrence. We did not observe any difference in sensory neuropathy occurrence between the reduced initial dose of chemotherapy (> 10% dose reduction) or standard dose (≤ 10% dose reduction) in either of the cohorts (Fig. 3A). ePRO follow-up was also able to detect higher grade (≥ 2) peripheral sensory neuropathy (Fig. 3B).

Next, we analyzed if there would be a difference between the cohorts in time to chemotherapy dose reduction, dose delays, or unplanned treatment termination. In dose reductions and dose delays, there was a non-significant tendency for later occurrence in the ePRO cohort. There was no difference between the cohorts in unplanned treatment terminations (Fig. 3C–E).

**Fig. 4:**
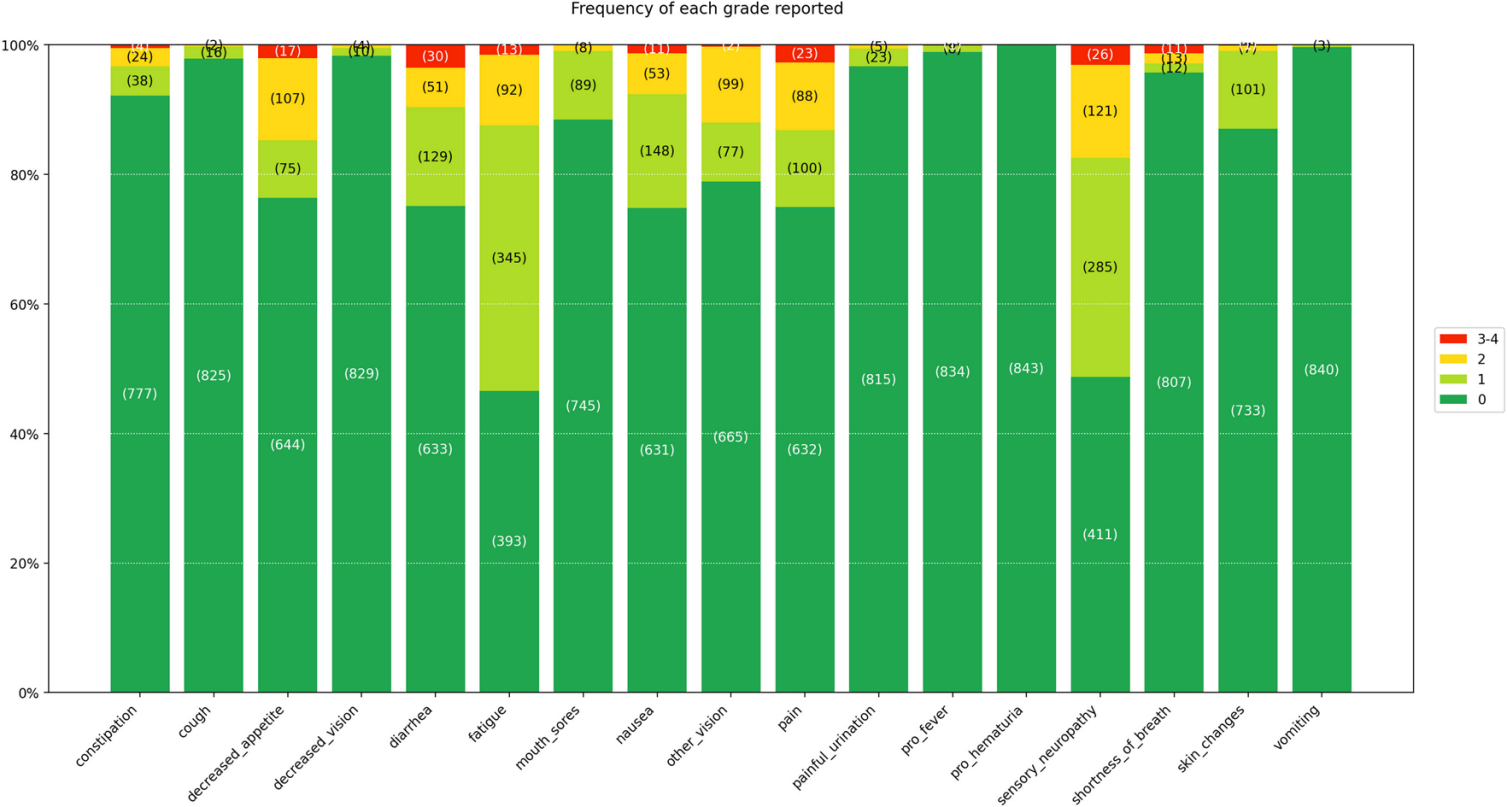
ePRO reported symptoms and their NCI-CTCAE grading

In the ePRO cohort, 17 symptoms were registered in all together 843 questionnaires. Of the recorded symptoms, fatigue (53%) and peripheral sensory neuropathy (51%) were the most frequent. Of the grade 3–4 symptoms, diarrhea (4%) and peripheral sensory neuropathy (3%) were the most common (Fig. 4).

### Disease-free and overall survival

**Fig. 5:**
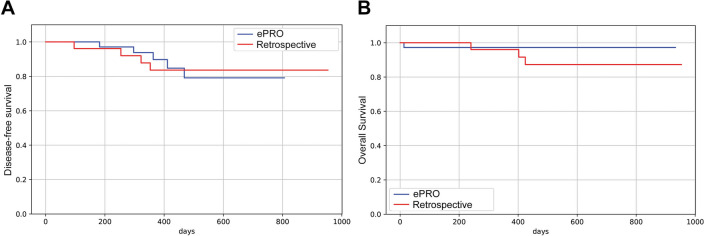
Disease-free (DFS) and overall survival (OS) in ePRO (blue) vs. retrospective cohorts (red). **A** Kaplan–Meier analysis for DFS. **B** Kaplan–Meier analysis for OS

Disease-free (DFS) and overall survival (OS) analysis was carried out. The analysis was done by comparing DFS between the retrospective and prospective ePRO cohorts. We could not detect any difference in DFS or OS (Fig. 5A, B).

## Discussion

The current study investigated the feasibility of ePRO follow-up in CRC patients receiving oxaliplatin-based chemotherapy as an adjuvant treatment. In addition, we investigated a novel holistic approach using a decision support tool combining ePRO data and laboratory work to a semi-automated chemotherapy cycle prescription, personalized symptom management support, and messaging tool. We do believe that this combinatory approach could reduce the workload of healthcare unit, improve cancer care, and enhance patient empowerment. The main findings of our study are that the investigated approach is very easy to use as well as appealing for the patients and HCPs. Furthermore, the investigated tool was able to significantly reduce routine phone calls to patients and enhance the detection of peripheral sensory neuropathy.

ePRO follow-up coupled to urgency algorithms has been shown to improve the QoL and survival and reduce the number of unscheduled visits in cancer patients (Basch et al. 2015, 2017; Denis et al. 2017a, b). However, currently investigated tools have been very simplified with limited integrations. Patient-reported symptoms can provide valuable comprehensive and real-time information which has an important role in clinical decision-making (Trackovic-Vidakovic et al. 2012; Bennett et al. 2012; Benze et al. 2017; Cleeland et al. 2011). Integrating ePRO data to other health-related information could increase their impact. We vision that combining multiple health information sources to an automated/semi-automated approach could generate digital care pathways and surpass the impact of sole ePRO follow-up. Of notice, our approach was able to reduce healthcare workload by decreasing phone calls.

Based on patient experience survey, the feasibility of ePRO tool was on excellent level. Almost all patients reported that the use of the platform was easy, and they felt that the tool improved their cancer care. Since cancer patients are generally older with co-morbidities, and their performance may be impaired by symptoms, user-friendliness and the sense of importance are essential (Laugsand et al. 2010; Reilly et al. 2013; Valderas et al. 2008; Velikova et al. 2010).

ePRO tool was able to detect sensory peripheral neuropathy, one of the most relevant oxaliplatin-related AEs, significantly earlier than seen in the retrospective cohort. This, however, did not lead to earlier dose reduction, delays, or treatment discontinuations. There is no clinically uniform guidance on oxaliplatin dose reduction or dose delay implementation on NCI-CTCAE-based grading of peripheral sensory neuropathy and, therefore, this mainly relies on HCP preference and experience. We speculate that even though sensory neuropathy was detected earlier, HCPs felt that its severity would not require dose alterations. Having specific CTCAE-based guidance on dose alterations would probably result in prompt actions.

Our ePRO cohort was generally dosed with higher initial chemotherapy dose compared to the retrospective cohort, so it was somewhat surprising that chemotherapy dose reduction, delays, or treatment discontinuations were not seen more frequently. In the trial, ePRO followed patients received symptom-based digital supportive and/or self-management feedback. One could speculate that this feedback could have supported in symptom management and chemotherapy adherence.

Our study has some limitations. The study was generally limited in number of subjects and the current report focused only on the adjuvant treated. We did some comparison of ePRO followed to a retrospective cohort of patients collected from the same institutes. This approach has obvious uncertainties and does not supplement for a true randomized trial. Furthermore, limited data were available on retrospective patients compared to the prospective cohort.

In conclusion, the current study investigated a decision support tool combining ePRO data and laboratory work to a semi-automated chemotherapy cycle prescription, personalized symptom management support, and messaging tool in CRC patient receiving oxaliplatin-based adjuvant chemotherapy. The study results suggest that the approach is feasible from the perspectives of patients and HCP and streamlines the workflow in the care unit. Furthermore, the ePRO monitoring detects evolving neuropathy earlier, which may enhance symptom management and improve the quality in cancer care.

## Data Availability

The datasets generated and/or analyzed during the current study are not publicly available but are available from the corresponding author on reasonable request.
